# Supplementary material for: Polyketide synthase gene clusters in the ericoid mycorrhizal fungus Oidiodendron maius: genome-wide prediction, expression profiling, and functional characterization of knockout mutants
Source: Front Plant Sci. 2026 Sep 16;17:1924430. doi: 10.3389/fpls.2026.1924430 (PMC13623903; doi:10.3389/fpls.2026.1924430)
Supplement: Supplementary file 1 [file SupplementaryFile1.zip › Supplementary Materials/Supplementary Folder 1.zip!/Oidma1_AssemblyScaffolds_Repeatmasked/knownclusterblast/cluster27/jgi.p_Oidma1_181890_mibig_hits.html]

| MIBiG Protein | Description | MIBiG Cluster | MiBiG Product | % ID | % Coverage | BLAST Score | E-value |
| --- | --- | --- | --- | --- | --- | --- | --- |
| ACB12564.1 | Fum19 | BGC0000063 | Fumonisin\_biosynthetic\_gene\_cluster | 41 | 103.41997264 | 1032 | 6.3e-301 |
| AAN74822.1 | Fum19p | BGC0000062 | Fumonisin\_biosynthetic\_gene\_cluster | 40 | 102.530779754 | 1001 | 9.1e-292 |
| AMM63163.1 | AniL | BGC0001371 | Echinocandin\_B\_biosynthetic\_gene\_cluster | 35 | 101.915184679 | 767 | 2.2e-221 |
| AFT91383.1 | EcdL | BGC0000338 | Echinocandin\_B\_biosynthetic\_gene\_cluster | 35 | 103.146374829 | 739 | 8.4e-213 |
| EPE34342.1 | P-loop\_containing\_nucleoside\_triphosphate\_hydrolase | BGC0001035 | Pneumocandin\_biosynthetic\_gene\_cluster | 34 | 92.8180574555 | 728 | 1.5e-209 |
| AMR44284.1 | ABC\_transporter | BGC0001398 | Phomopsins\_biosynthetic\_gene\_cluster | 30 | 105.60875513 | 581 | 2.2e-165 |
| OAQ83754.1 | ABC\_multidrug\_transporter | BGC0001358 | Leucinostatins\_biosynthetic\_gene\_cluster | 36 | 61.4911080711 | 517 | 4e-146 |
| CRG85577.1 | hypothetical\_protein | BGC0001402 | Cyclochlorotine\_biosynthetic\_gene\_cluster | 25 | 103.830369357 | 324 | 4.9e-88 |
| AAS92552.1 | SirA | BGC0001044 | Sirodesmin\_biosynthetic\_gene\_cluster | 25 | 62.585499316 | 129 | 4e-29 |
| ctg1\_orf5 |  | BGC0000321 | Chaetocin\_biosynthetic\_gene\_cluster | 23 | 68.1258549932 | 126 | 3.4e-28 |
| CAI47637.1 | putative\_ABC-type\_aminoglycoside\_exporter | BGC0000692 | Apramycin\_biosynthetic\_gene\_cluster | 26 | 35.1573187415 | 119 | 2.4e-26 |
| BAI23328.1 | putative\_ABC-transporter | BGC0000949 | A-500359s\_biosynthetic\_gene\_cluster | 30 | 29.8905608755 | 116 | 2.7e-25 |
| BAJ19059.1 | putative\_ABC\_transporter | BGC0000288 | A-503083\_biosynthetic\_gene\_cluster | 28 | 30.7113543092 | 113 | 3e-24 |
| AAK73499.1 | AmphH | BGC0000015 | Amphotericin\_biosynthetic\_gene\_cluster | 28 | 27.0861833105 | 111 | 8.6e-24 |
| EHQ60564.1 | ABC\_transporter | BGC0001356 | Paeninodin\_biosynthetic\_gene\_cluster | 26 | 23.9398084815 | 108 | 9.5e-23 |
| CBW74823.1 | Multidrug\_resistance\_ABC\_transporter\_ATP-binding\_and\_permease\_protein | BGC0000571 | Burhizin\_biosynthetic\_gene\_cluster | 26 | 41.1764705882 | 108 | 9.5e-23 |
| EEM14530.1 | hypothetical\_protein | BGC0001210 | Pseudomycoicidin\_biosynthetic\_gene\_cluster | 26 | 29.2749658003 | 106 | 2.1e-22 |
| ALX37944.1 | GaeT | BGC0001388 | Gassericin\_E\_biosynthetic\_gene\_cluster | 22 | 31.805745554 | 106 | 3.6e-22 |
| BAM09402.1 | putative\_ABC-transporter | BGC0000619 | Gassericin\_T\_biosynthetic\_gene\_cluster | 22 | 31.805745554 | 105 | 4.7e-22 |
| AAG28766.1 | CoaD | BGC0000617 | Coagulin\_biosynthetic\_gene\_cluster | 26 | 22.5718194254 | 105 | 6.2e-22 |
| ACA97578.1 | PmxC | BGC0000408 | Polymyxin\_biosynthetic\_gene\_cluster | 27 | 29.8221614227 | 104 | 1.1e-21 |
| ANP43732.1 | ThsT | BGC0001391 | Thusin\_biosynthetic\_gene\_cluster | 21 | 41.1080711354 | 103 | 2.3e-21 |
| AEZ51518.1 | PmxC | BGC0001153 | Polymyxin\_biosynthetic\_gene\_cluster | 27 | 29.8221614227 | 102 | 3.1e-21 |
| CAB38879.1 | putative\_ABC\_transporter | BGC0000315 | Calcium-dependent\_antibiotic\_biosynthetic\_gene\_cluster | 26 | 28.317373461 | 102 | 4e-21 |
| BAE07080.1 | possible\_ABC\_transporter | BGC0000693 | Butirosin\_biosynthetic\_gene\_cluster | 23 | 40.9712722298 | 101 | 6.8e-21 |
| AHF21226.1 | TriB | BGC0000449 | Tridecaptin\_biosynthetic\_gene\_cluster | 30 | 20.0410396717 | 101 | 1.2e-20 |
| BAE56595.1 |  | BGC0001123 | Aspirochlorine\_biosynthetic\_gene\_cluster | 26 | 21.4090287278 | 100 | 1.5e-20 |
| WP\_010071619.1 | ABC\_transporter\_ATP-binding\_protein | BGC0000578 | SRO15-2005\_biosynthetic\_gene\_cluster | 26 | 33.1737346101 | 100 | 1.5e-20 |
| WP\_043270371.1 | ABC\_transporter\_ATPase | BGC0001188 | Legonaridin\_biosynthetic\_gene\_cluster | 24 | 38.7824897401 | 100 | 2e-20 |
| AJM89736.1 | PmxC | BGC0001192 | Colistin\_biosynthetic\_gene\_cluster | 26 | 30.1641586867 | 99 | 3.4e-20 |
| ALV82355.1 | ABC\_transporter | BGC0001370 | Lipopeptide\_8D1-1\_/\_lipopeptide\_8D1-2\_biosynthetic\_gene\_cluster | 25 | 28.317373461 | 98 | 7.5e-20 |
| AAX31531.1 | putative\_ABC\_transporter | BGC0000336 | Daptomycin\_biosynthetic\_gene\_cluster | 24 | 36.9357045144 | 97 | 9.8e-20 |
| WP\_006126653.1 | ABC\_transporter | BGC0000554 | SRO15-3108\_biosynthetic\_gene\_cluster | 28 | 21.7510259918 | 97 | 1.3e-19 |
| ALE27499.1 | ABC\_transporter | BGC0001292 | A33853\_biosynthetic\_gene\_cluster | 27 | 21.9562243502 | 94 | 1.1e-18 |
| CAJ88629.1 | putative\_netropsin\_resistance\_protein\_subunit\_2 | BGC0000327 | Congocidine\_biosynthetic\_gene\_cluster | 24 | 34.8153214774 | 94 | 1.1e-18 |
| ABQ84449.1 | salivaricin\_ABC-transporter | BGC0000624 | Salivaricin\_CRL1328\_alpha\_peptide\_/\_salivaricin\_CRL1328\_beta\_peptide | 22 | 31.8741450068 | 94 | 1.1e-18 |
| CAA11793.1 | PCZA363.2 | BGC0000322 | Chloroeremomycin\_biosynthetic\_gene\_cluster | 28 | 20.2462380301 | 93 | 1.9e-18 |
| BAB04170.1 | lantibiotic\_mersacidin\_transporter\_system | BGC0000517 | Haloduracin\_alpha\_/\_haloduracin\_beta\_biosynthetic\_gene\_cluster | 24 | 46.9904240766 | 92 | 4.1e-18 |
| ADI10107.1 | cytolysin\_B\_transport\_protein | BGC0001229 | SBI\_06990\_alpha\_/\_SBI\_06989\_beta\_biosynthetic\_gene\_cluster | 26 | 26.3337893297 | 92 | 4.1e-18 |
| AHJ59536.1 | ABC\_transporter\_protein | BGC0000502 | Cerecidin\_/\_cerecidin\_A1\_/\_cerecidin\_A2\_/\_cerecidin\_A3\_/\_cerecidin | 24 | 30.5061559508 | 91 | 7e-18 |
| EFI65097.1 | lantibiotic\_transport\_protein | BGC0001407 | Bicereucin\_biosynthetic\_gene\_cluster | 23 | 36.3201094391 | 91 | 7e-18 |
| AAP57696.1 | putative\_secretion\_protein | BGC0000768 | Gellan\_polysaccharide\_biosynthetic\_gene\_cluster | 27 | 23.3242134063 | 91 | 1.2e-17 |
| AAC44070.1 | unknown | BGC0000797 | Sphingan\_polysaccharide\_biosynthetic\_gene\_cluster | 27 | 23.3242134063 | 90 | 2.1e-17 |
| ABW74893.1 | ABC\_transporter | BGC0000759 | Diutan\_polysaccharide\_biosynthetic\_gene\_cluster | 27 | 23.3242134063 | 89 | 4.6e-17 |
| AHF21240.1 | CrnT | BGC0000500 | Carnolysin\_A1\_/\_carnolysin\_A2\_biosynthetic\_gene\_cluster | 29 | 25.0341997264 | 87 | 1.3e-16 |
| EFY94505.1 | multidrug\_resistance\_protein | BGC0000337 | Destruxins\_biosynthetic\_gene\_cluster | 26 | 30.9165526676 | 87 | 1.3e-16 |
| KIH99790.1 | hypothetical\_protein | BGC0001176 | Streptomonomicin\_biosynthetic\_gene\_cluster | 26 | 23.3242134063 | 87 | 1.7e-16 |
| XP\_011392729.1 | ABC\_transporter | BGC0001281 | Ustilagic\_acid\_biosynthetic\_gene\_cluster | 25 | 38.5088919289 | 86 | 3e-16 |
| AFS60105.1 | PaenT | BGC0000540 | Paenibacillin\_biosynthetic\_gene\_cluster | 23 | 41.1080711354 | 86 | 3.9e-16 |
| AHZ61877.1 | Lom43 | BGC0000240 | Lomaiviticin\_biosynthetic\_gene\_cluster | 23 | 40.218878249 | 85 | 5.1e-16 |
| BAU50927.1 | ABC\_transporter | BGC0001379 | BD-12\_biosynthetic\_gene\_cluster | 24 | 35.1573187415 | 84 | 8.6e-16 |
| CAI47638.1 | putative\_ABC-type\_aminoglycoside\_exporter | BGC0000692 | Apramycin\_biosynthetic\_gene\_cluster | 25 | 27.2229822161 | 84 | 1.1e-15 |
| ABP54659.1 | ABC\_transporter\_related | BGC0000241 | Lomaiviticin\_biosynthetic\_gene\_cluster | 22 | 39.9452804378 | 83 | 1.9e-15 |
| AAZ76601.1 | BhtT | BGC0000497 | BhtA1\_/\_BhtA2\_biosynthetic\_gene\_cluster | 24 | 30.9165526676 | 83 | 2.5e-15 |
| BAD72775.1 |  | BGC0000552 | Smb\_A\_/\_Smb\_B\_biosynthetic\_gene\_cluster | 23 | 32.8317373461 | 82 | 3.3e-15 |
| CAD15511.1 | probable\_composite\_atp-binding\_transmembrane\_abc\_transporter\_protein | BGC0001014 | Micacocidin\_biosynthetic\_gene\_cluster | 25 | 33.0369357045 | 82 | 3.3e-15 |
| AAF71764.1 | NysH | BGC0000115 | Nystatin\_biosynthetic\_gene\_cluster | 24 | 35.4309165527 | 81 | 1.2e-14 |
| CBL93714.1 | ABC\_multidrug\_transporter | BGC0000360 | GE81112\_biosynthetic\_gene\_cluster | 23 | 36.0465116279 | 78 | 8.1e-14 |
| AAA62651.1 |  | BGC0000504 | Cytolysin\_ClyLl\_/\_cytolysin\_ClyLs\_biosynthetic\_gene\_cluster | 23 | 30.779753762 | 78 | 8.1e-14 |
| ALR96371.1 | lantibiotic\_ABC\_transporter | BGC0001289 | Penisin\_biosynthetic\_gene\_cluster | 25 | 25.6497948016 | 77 | 1.8e-13 |
| BAI23327.1 | putative\_ABC-transporter | BGC0000949 | A-500359s\_biosynthetic\_gene\_cluster | 25 | 30.9165526676 | 74 | 1.2e-12 |
| AHH99931.1 | ABC\_transporter\_protein | BGC0000002 | Aculeximycin\_biosynthetic\_gene\_cluster | 24 | 30.1641586867 | 72 | 4.4e-12 |
| CAH94306.1 | putative\_ABC\_transporter\_StrW | BGC0000717 | Streptomycin\_biosynthetic\_gene\_cluster | 23 | 35.1573187415 | 71 | 9.9e-12 |
| BAG22745.1 | putative\_ABC-type\_multidrug\_transport\_system\_ATPase\_and\_permease\_component | BGC0000724 | Streptomycin\_biosynthetic\_gene\_cluster | 23 | 35.1573187415 | 71 | 9.9e-12 |
| AHF21235.1 | PabT | BGC0000542 | Paenicidin\_B\_biosynthetic\_gene\_cluster | 25 | 24.6922024624 | 68 | 8.3e-11 |
| CAA07371.1 | StrW | BGC0000690 | 5'-hydroxystreptomycin\_biosynthetic\_gene\_cluster | 22 | 34.6785225718 | 68 | 8.3e-11 |
| CAM02315.1 | putative\_ABC\_transporter\_transmembrane | BGC0000349 | Erythrochelin\_biosynthetic\_gene\_cluster | 22 | 35.2941176471 | 68 | 8.3e-11 |
| WP\_020635023.1 | multidrug\_ABC\_transporter\_permease | BGC0001211 | Albachelin\_biosynthetic\_gene\_cluster | 22 | 33.926128591 | 67 | 1.4e-10 |
| BAL72550.1 | lariatin\_biosynthetic\_protein | BGC0000575 | Lariatin\_biosynthetic\_gene\_cluster | 23 | 33.1053351573 | 66 | 3.2e-10 |
| CBG75489.1 | putative\_ABC-transport\_system\_ATP-binding\_component | BGC0000423 | Scabichelin\_biosynthetic\_gene\_cluster | 22 | 34.268125855 | 66 | 4.1e-10 |
| CBL93713.1 | ABC\_multidrug\_transporter | BGC0000360 | GE81112\_biosynthetic\_gene\_cluster | 23 | 38.3720930233 | 62 | 3.5e-09 |
| CAB53323.1 | putative\_ABC-transporter\_transmembrane\_protein | BGC0000325 | Coelichelin\_biosynthetic\_gene\_cluster | 23 | 36.9357045144 | 60 | 1.7e-08 |
| CBG75488.1 | putative\_ABC\_transport\_system\_integral\_membrane\_component | BGC0000423 | Scabichelin\_biosynthetic\_gene\_cluster | 23 | 36.0465116279 | 60 | 2.3e-08 |
| CAM02316.1 | ABC\_transporter\_protein,\_ATP-binding\_component | BGC0000349 | Erythrochelin\_biosynthetic\_gene\_cluster | 23 | 37.5512995896 | 57 | 1.1e-07 |
| AAC72259.1 | LctT | BGC0000521 | Lacticin\_481\_biosynthetic\_gene\_cluster | 23 | 20.9986320109 | 57 | 1.1e-07 |
| BAC98761.2 | NukT | BGC0000539 | Nukacin\_ISK-1\_biosynthetic\_gene\_cluster | 21 | 22.1614227086 | 52 | 4.7e-06 |
| ABI30230.1 | McdT | BGC0000526 | Macedocin\_biosynthetic\_gene\_cluster | 24 | 29.0697674419 | 51 | 8.1e-06 |
